# Advanced Research in Porcine Reproductive and Respiratory Syndrome Virus Co-infection With Other Pathogens in Swine

**DOI:** 10.3389/fvets.2021.699561

**Published:** 2021-08-26

**Authors:** Dengshuai Zhao, Bo Yang, Xingguo Yuan, Chaochao Shen, Dajun Zhang, Xijuan Shi, Ting Zhang, Huimei Cui, Jinke Yang, Xuehui Chen, Yu Hao, Haixue Zheng, Keshan Zhang, Xiangtao Liu

**Affiliations:** State Key Laboratory of Veterinary Etiological Biology, National Foot-and-Mouth Disease Reference Laboratory, Lanzhou Veterinary Research Institute, Chinese Academy of Agriculture Science, Lanzhou, China

**Keywords:** porcine reproductive and respiratory syndrome virus, porcine respiratory disease complex, co-infection, secondary infection, pathogens

## Abstract

The porcine reproductive and respiratory syndrome virus (PRRSV) is the pathogen causing epidemics of porcine reproductive and respiratory syndrome (PRRS), and is present in every major swine-farming country in the world. Previous studies have demonstrated that PRRSV infection leads to a range of consequences, such as persistent infection, secondary infection, and co-infection, and is common among pigs in the field. In recent years, coinfection of PRRSV and other porcine pathogens has occurred often, making it more difficult to define and diagnose PRRSV-related diseases. The study of coinfections may be extremely suitable for the current prevention and control in the field. However, there is a limited understanding of coinfection. Therefore, in this review, we have focused on the epidemiology of PRRSV coinfection with other pathogens in swine, both *in vivo* and *in vitro*.

## Introduction

The porcine reproductive and respiratory syndrome virus (PRRSV) is a single-stranded positive-sense RNA virus with a capsid, and it belongs to the family *Arteriviridae* (1). The PRRSV genome is ~15 kb in length and contains at least 10 open reading frames (ORFs). ORF1a and ORF1b encode RNA replicase and at least 16 non-structural proteins. ORF2–ORF7 encode glycoproteins (GP2–GP5), membrane protein M, and nucleocapsid protein N (2, 3). Porcine reproductive and respiratory syndrome (PRRS) caused by PRRSV, also known as porcine blue ear disease, is a highly immunosuppressive disease (4, 5). It causes subclinical, lethal, and persistent infections (6). Clinical symptoms of PRRS depend on the infecting strain, growth stage, immune status, secondary or co-infecting pathogens, environmental conditions, and disease management level (7).

In 1987, PRRSV first became prevalent in the United States, and then gradually appeared in swine-farming countries worldwide (8). It was described with the following two genotypes: PRRSV-1 (alternatively Betaarterivirus suid 1), of European origin, and PRRSV-2 (alternatively Betaarterivirus suid 2) of North American origin (9). The prevalent PRRSV strains in China are mainly the PRRSV-2. Although the outbreak of the African swine fever virus (ASFV) in 2018 masked PRRS, it is still difficult to get rid of completely (10, 11). For this reason, it is necessary to understand the evolutionary trends of PRRSV in China.

The disease was initially reported in China in 1996, and since then the highly pathogenic PRRSV (HP-PRRSV) strain has caused outbreaks in several provinces and led to serious economic losses (12, 13). After 2012, the new recombinant strains GM2 and NADC30-like appeared in China (14, 15). After 2015, HP-PRRSV and NADC30-like became prevalent (13). The emergence of NADC30-like strains may be due to the recombination of North American NADC30 strains and Chinese HP-PRRSV strains (16). Although not as highly pathogenic as HP-PRRSV, NADC30-like strains are known for their high incidence of recombination with other viral strains, resulting in altered virulence. Moreover, there might be outbreaks of NADC30-like strain in vaccinated swine (17). Furthermore, coinfection of NADC30-like strains and HP-PRRSV is common in piglets with porcine respiratory disease complex (PRDC) (18). It is demonstrated that recombination caused by coinfection with different strains or genotypes makes the epidemiology of PRRSV strains more complex and diverse. Moreover, this may be one of the reasons for the different results of PRRSV coinfection with other pathogens. Therefore, it is necessary to review the prevalence of PRRSV.

Coinfection has become a common phenomenon in current swine farms, and PRRSV is one of the key pathogens (19). The PRRSV coinfection viruses include porcine circovirus 2 (PCV2), pseudorabies virus (PRV), swine influenza virus (SIV), classical swine fever virus (CSFV), hepatitis E virus (HEV), porcine parvovirus (PPV), porcine group A rotavirus (PARV) and porcine epidemic diarrhea virus (PEDV) (20, 21). PRRSV can cause co-infection/secondary infection with bacteria such as *Haemophilus parasuis* (HPS), *Streptococcus suis* (SS), and *Actinobacillus pleuropneumoniae* (APP) (22–24). Moreover, coinfection of PRRSV and *Toxoplasma gondii* (T. gondii) or *Mycoplasma hyopneumoniae* (Mhp) often occurs (25, 26). However, there is a limited understanding of it. The current research is mainly on the interaction between host and pathogens, which limits the study of coinfection (19). Therefore, in this review, we discuss the interaction of PRRSV and the above-mentioned pathogens as systematically as possible. The aim is to provide a reference for the prevention and control of related diseases, the production of vaccines, and research methods for coinfection.

## Co-infection of PRRSV and Viruses

Although coinfection is important in this field, there are limited studies on coinfection of PRRSV and other viruses. The current single host-pathogen research method and the extremely high mutation rate of PRRSV itself may be the factors that limit the study of coinfection. Moreover, in limited coinfection experimental studies, different PRRSV strains, infection sequence, animal models, and susceptible cells affect the observation results (Table 1).

**Table 1:** Co-infection of PRRSV with other viruses.

**Coinfections**	**Protocols**	**Selected cells or Swine**	**Observations**	**References**
PRRSV PCV2	PRRSV-2: JS-1 PCV2: Local strain PRRSV then PCV2 *In vitro*	Cells: PAM	- PRRSV and PCV2 replication Enhanced - The coinfection induced IκBα degradation and Phosphorylation - NF-κB signaling pathway is activated	(27)
	PRRSV-2: HP-PRRSV HBR PCV2: PCV2b YJ PRRSV then PCV2 PCV2 then PRRSV Simultaneous infection *In vivo*/vitro	Cells: MARC-145 Cells: PK-15 Swine: 35-day-old piglets	- Both virus replication is promoted - The levels of TNF-α and IL-10 increased - The number of CD4+ and NK cells decreased - CD8+ cells also increased - The coinfection Caused serious clinical symptoms and lesions	(28)
	PRRSV-2: ATCC VR2385 PCV2: PCV2b NC-16845 Simultaneous infection *In vivo*	Swine: 2- to 6-week-old pigs(SPF)	- PCV2 replication promoted by PRRSV - PRRSV infection prolonged the existence of PCV2 in serum and body fluids	(29)
PRRSV PRV	PRRSV-2: SDRPI5D PRV: AUJII3K PRRSV then PRV PRV then PRRSV *In vivo*	Swine: 8-week-old pigs(SPF)	- A better secondary antibody response produced by the coinfection	(30)
PRRSV CSFV	PRRSV-2: JXwn06 PRRSV-2: H-B1/3.9 PRRSV-2: CHsx1401 CSFV: CSFV-C PRRSV then CSFV *In vitro*	Cells: PAM39	- The level of pro-inflammatory cytokines is up-regulated, especially TNF-α. - Replication of CSFV is suppressed by PRRSV.	(31)
PRRSV SIV	PRRSV-1: Lelystad SIV:H1N1A Simultaneous infections *In vivo*	Swine: 10-week-old piglets (SPF)	- The coinfection Caused serious clinical symptoms - SIV replication is hardly interfered by PRRSV	(32)
	PRRSV-1: Lelystad SIV: H3N2 PRRSV then SIV *In vivo*	Swine: 3-week-old piglets (SPF)	- The coinfection caused severe bronchiolar wall inflammation - Previous PRRSV infection has no effect on the SIV infection stage	(33)
	PRRSV-2: ISU-12-SAH SIV: H1N1 Sk02 SIV then PRRSV *In vitro*	Cells: PAM	- SIV replication is hardly interfered by PRRSV - The levels of INF-β, TNF-α and IL-10 increased mRNA levels of cytokines were additive effect	(34)
PRRSV HEV	PRRSV-1: PRRS-2005-29-24-1 HEV3: FR-SHEV3e Simultaneous infection *In vivo*	Swine: 5-week-old piglets (SPF)	- The shedding of HEV increased significantly, and prolonged significantly - Viraemia occurred earlier and longer	(35, 36)

### Co-infection of PRRSV and PCV2

PCV is a naked, circular, single-stranded DNA virus belonging to the family *Circoviridae*. It can be divided into four types: PCV1, PCV2, PCV3, and PCV4 (37, 38). It is the main pathogen causing porcine circovirus diseases (PCVDs) and porcine circovirus 2-systemic disease (PCV2-SD) (39, 40). A series of disease syndromes caused by PCV2 are collectively known as porcine circovirus-associated disease (PCVAD) (41). Its symptoms mainly include PCV2-associated respiratory disease, low growth performance, and postweaning multisystemic wasting syndrome (PMWS) (42, 43). Samples of 159 sick and dead swine collected from eight provinces and cities in China were evaluated, and it was demonstrated that the coinfection rates of PRRSV, CSFV, PCV2, and PCV3 reached 15.72%. Meanwhile, the coinfection rate of PRRSV and PCV2 reached 10.69% (44). Another study on lesions of proliferative and necrotizing pneumonia (PNP) showed that coinfection with PRRSV and PCV2 occurred more often in cases, including in weaned piglets, with a coinfection rate of 42% (45). Furthermore, PRRSV was detected in 51.9% of the PMWS cases (46). A high rate of coinfection involving PRRSV and PCV2 was observed in the swineherd where the lesions occurred. Therefore, it is assumed that coinfection of PRRSV and PCV2 may be one of the main causes of PCVAD.

Both PRRSV and PCV2 target the host's immune cells, disrupting their immune function and leading to increased susceptibility to primary and secondary pathogens, which may affect host growth performance and the incidence and lethality of associated diseases (47). It has been reported that the inoculation of PCV2 1 week after HP-PRRSV resulted in the highest viral load, the most severe clinical signs, and the highest mortality (60%) in piglets (28). In further studies, it was found that a PCV2 infection in swine infected with HP-PRRSV enhanced the replication of both viruses and led to more severe clinical signs and lesions (28). Furthermore, this result seems to be verified in an *in vitro* coinfection model, based on changes in the levels of PRRSV N gene and PCV2 Cap gene in porcine alveolar macrophage (PAM) cells. It was concluded that the coinfection model enhanced the replication of PRRSV and PCV2 (27). Moreover, the levels of p-IκBα and p65 proteins in the nucleus were significantly increased after coinfection. The co-infection could induce NF-κB translocation from the cytoplasm to nucleus, which leads to activation of the NF-κB pathway (27). Cumulatively, these results demonstrate synergistic effects during PRRSV and PCV2 coinfection (28, 48).

Based on the Cap gene sequence of PCV2, five subtypes can be classified: PCV2a, PCV2b, PCV2c, PCV2d, and PCV2e (49). Some studies have reported that PRRSV can also affect infection with PCV2 subtypes (mainly PCV2a and PCV2b) by prolonging viremia and *in vivo* shedding of PCV2 (29). Coinfection of different subtypes of PCV2 with PRRSV also increased the mutation rate of PRRSV in successive generations. The mutation rates of ORF5 and ORF6 in swine co-infected with PRRSV and PCV2b were significantly higher than in those infected with PRRSV alone. The ORF7 mutation rate in swine coinfected with PRRSV and PCV2a was also significantly higher (50). Therefore, it is shown that coinfection with different subtypes of PRRSV and PCV2 can cause a higher mutation rate of PRRSV, which may be one of the reasons for difficulty in complete clearance of PRRSV.

### Co-infection of PRRSV and PRV

PRV is a linear, double-stranded DNA virus with an envelope, also known as porcine herpesvirus type I, belonging to the family *Herpesviridae* (51). PRV causes neurological and febrile symptoms and is one of the major causes of infectious diseases affecting the global pig industry (52). It has an excessively broad host range, infecting both domestic and wild animals, and causes different symptoms (53). Infected animals typically develop fever, sneezing, coughing, and vomiting. This is occasionally accompanied by typical neurological signs such as twitching, aggressiveness, and lack of coordination. PRV can infect swine of all ages, with a 100% lethality rate in piglets up to 15 days of age (54). However, the adult tolerant swine can establish and maintain latent infection in the peripheral nervous system. In the event of external stimulation or immune deficiency, the latently infected viral genome can be reactivated. This causes massive viral replication in epithelial tissues and outgrowth to other tissues, as well as outgrowing the virus, making the host a dangerous source of infection again (55).

Previous surveys have also shown a high proportion of latent PRV infections in swine (53). It has been found that the current cause of infection in swine farms is the continuous mutation of PRV strains. Although the traditional Bartha-K61 vaccine provides complete protection against the classical strain (SC), it does not protect against the currently prevalent mutated strain (HeN1) (56). Co-infection of PRRSV and PRV is also common, with a co-infection rate of 36% (21). Moreover, it is common in intensive fattening swine in Japan (57). However, there are few studies on the co-infection of the two viruses *in vivo* and *vitro*, and the interaction between them needs to be further explored. Moreover, the latent infection of PRV in adult-tolerant pigs may be one of the reasons for the high coinfection rate of the two viruses.

### Co-infection of PRRSV and CSFV

CSFV, a single-stranded positive-sense RNA virus with a capsid belongs to the *Flaviviridae* family, is the pathogen that causes classical swine fever (58). It has been suggested that CSFV includes three different subtypes; however there is no international consensus on their classification system (59). CSFV contains a large ORF, 3′-UTR and 5′-UTR, encoding four structural proteins: C, Erns, E1, and E2. It also encodes eight nonstructural proteins: Npro, P7, NS2, NS3, NS4A, NS4B, NS5A, and NS5B (60). Established multiplex real time PCR (MRT-PCR) was used to analyze 69 clinical samples from three provinces in China, showing that the coinfection rate of PRRSV and CSFV reached 4.4% (20). Overall, the coinfection rate of PRRSV and CSFV in China is about 0–7.7% (20). Furthermore, PRRSV infection affects CSFV vaccination. The Chinese (C) strain vaccine is considered to be the most effective in inducing protective immunity against CSFV (61), but in the field, immunization often fails owing to infection by immunosuppressive pathogens such as PRRSV.

It is clear that the coinfection rate of PRRSV and CSFV in intensive swine farms is low, but PRRSV infection in the field can cause CSFV vaccination failure. Moreover, current vaccination strategies against both viruses still use two single modified live vaccines, leading to mutual interference (62). Therefore, it is speculated that the PRRSV strain interacts with the CSFV vaccine strains. In a study, it was found that PRRSV infection caused the upregulation of pro-inflammatory cytokines *in vitro*, particularly TNF-α. The results showed that the replication of CSFV-C was inhibited, which also provided an explanation for the failure of CSFV vaccination caused by PRRSV (31). However, this study does not have the data of *in vivo* experiments. Moreover, the cell used by authors is PAM39, a cell line based on 3D4/21. This is not the primary culture cell. It is still difficult to choose a model suitable for the study of coinfection. Therefore, the absence of suitable host cells limits its study.

### Co-infection of PRRSV and SIV

SIV is any strains of the influenza virus family prevalent in swine, including influenza C and subtypes of influenza A, namely H1N1, H1N2, H2N1, H2N3, H3N1, and H3N2 (63, 64). Among these, the H1N1 SIV type A is an influenza virus with pandemic potential (65). Both SIV and PRRSV can cause subclinical infection; the symptoms of infection in swine mainly include high temperature, fever, cough, and dyspnea, which are difficult to distinguish clinically (66–68). Therefore, coinfection caused by SIV and PRRSV is even more difficult to prevent and control.

In a study involving 636 SIV-positive cases, 109 PRRSV-positive samples were detected, and the coinfection rate reached 17% (69). The study on coinfection of PRRSV and SIV was reported as early as 1996. The results showed that the coinfection group had more severe clinical symptoms and growth retardation than the single infection group. However, there was little effect on SIV replication (32). This is consistent with the results of another study which showed that the coinfection group suffered more severe inflammation of the bronchial wall and that preinfection with PRRSV did not affect the stage of SIV infection (33). In contrast, a different clinical outcome was found in another study (70). The phenomenon may be related to different strains and the immune status of swine. Moreover, the growth kinetics of the two viruses were studied in PAM cells. Results showed that no matter which virus first infected, PRRSV or SIV could slightly inhibit the replication of the later infected virus (34). This is consistent with the results of *in vivo* experimental studies (32). Further, the mRNA levels of IFN-β, IL-10, and TNF-α were found to increase significantly after coinfection and had an additive effect (34). However, this study only involved assessment of the expression of host cytokines. Similarly, such defects are common in all coinfection studies. Moreover, this is limited by the current level of research. Therefore, a clear understanding of the pathogenic mechanism of single infection is necessary for the study of co-infection. The latest research showed that PRRSV-1 changes the relationship between SIV and its main target cells, and the interaction between them could also affect vaccination (71). This undoubtedly provides a new research method for studying the interaction between the two viruses, and also indicates the complexity of the interaction between the two viruses in coinfection. To sum up, it can be inferred that the results of coinfection are also related to other factors, such as different virus strains, sequence of infection and susceptible cells.

### Co-infection of PRRSV and Other Viruses

In addition to the above-mentioned viruses, coinfection could also occur between PRRSV and other viruses, such as HEV, PPV, PARV, and PEDV (36, 72). HEV is a non-enveloped single-stranded positive-sense RNA virus, which belongs to the *Hepeviridae* family (73). It can be classified in four different genotypes and 24 subtypes based on its nucleotide sequence (74). Its transmission is common between humans and pigs, in which genotypes three and four are found in both pigs and humans (75, 76). Human infections with HEV can cause acute liver failure or chronic infections (77). Although pigs infected with HEV have no pathogenicity, they may enhance the pathogenicity of other porcine viruses (77). Coinfection of HEV and PRRSV can lead to long-term shedding of HEV and even chronic infection. After coinfection with PRRSV and HEV, the shedding of HEV was delayed by 1.9 times, and the specific immune response was delayed by 1.6 times compared with that of HEV infection alone (35). It is suggested that PRRSV has an important effect on the infection dynamics of HEV. Moreover, in another study, it was found that viremia occurred earlier and longer after co-infection of HEV and PRRSV (36). Thus, this may indicate that coinfection regulates the duration of viremia in HEV. However, another study found that there was no significant correlation between PRRSV-positive and HEV-positive status in plasma, tonsil and cecal content samples (78).

PPV is an important virus causing reproductive disorders in pigs. With the development of molecular technology, the detection of several new types of PPV have increased in the past few years. Such as the porcine parvovirus type 2 (PPV2), also known as Cnvirus (CnP-PARV4), porcine parvovirus type 3 (PPV3), and porcine parvovirus type 4 (PPV4) (79). Moreover, many studies have proved the possibility of coinfection of emerging PPV and PCV2, especially in PRDC (80). Although coinfection between PRRSV and emerging PPV remains to be studied, PRRSV, as one of the key pathogens in PRDC, has a great possibility of co-infection. In a study on the co-infection of PRRSV and other pathogens in the intestinal tract, it was found for the first time that PRRSV and porcine group a rotavirus (PARV) were co-infected, up to 52.4%. Furthermore, the coinfection rate of PPV and PEDV reached 33.3%, and the coinfection rate of PRRSV with more than two viruses also reached 33.3% (72). However, there are few studies on the co-infection of PRRSV, PARV, and PEDV *in vivo* and *in vitro*. The current research can only prove the co-existence of these viruses in the host, but cannot show that there is a complex interaction between them.

Multiple infections are common in intensive pig farms in addition to single and co-infections of related pathogens (20, 44). However, studies on the interaction of PRRSV-associated multiple infections and their pathogenic mechanisms are rare. Most relevant studies were based on MRT-PCR methods constructed for multiple pathogens and remain only in the stage of diagnostic testing. Therefore, coinfection studies between two pathogens associated with PRRSV are becoming increasingly important, contributing to the study of PRRSV-associated multiple infections.

## Co-infection of PRRSV and Bacteria

It is well-known that viral infections can induce an ideal environment for bacterial secondary infection through different mechanisms, such as disruption of the epithelial barrier, modulation of the expression of receptors involved in bacterial adhesion, and alteration of the host immune response (81–83). In addition, the results of secondary infections caused by different PRRSV strains seem to be inconsistent, which creates a great deal of confusion in the study of PRRSV-bacterial coinfection (Table 2).

**Table 2:** Co-infection of PRRSV with bacteria.

**Bacterium**	**Protocols**	**Selected cells or Swine**	**Observations**	**Reference**
*Haemophilus Parasuis*	PRRSV-2: VR-2332 HPS5: 29755 PRRSV then HPS *In vivo*	Swine: 9- to 12-day-old piglets (SPF)	- The clinical symptoms of co-infection are not as serious as SS alone - Co-infection caused severe lung congestion	(84)
	PRRSV-1: CAPM V-490 HPS5: HP 132 PRRSV then HPS *In vitro*	Cells: PAM	- The gene expression of pro-inflammatory cytokines (TNF-α, IL-1β, IL-8) in PAM is increased by co-infection - IL-1β had an additive effect in coinfection - Simultaneous infection also had an additive effect on the expression of CD80 mRNA	(85)
	PRRSV-2: HuN4 HPS5: Nagasaki PRRSV then HPS Simultaneous infection *In vivo*/vitro	Cells: PAM Swine: 4-week-old piglets (SPF)	- A higher bacterial load was observed in the lungs - HPS RNA enhanced PRRSV infection-mediated inflammatory responses - A synergistic effect between the HPS RNA and PRRSV	(23)
*Streptococcus suis*	PRRSV-2: VR-2385 SS2: ISU VDL 40634/94 PRRSV then SS *In vivo*	Swine: 3-week-old piglets (SPF)	- The proliferation of SS is promoted by PRRSV - Co-infection caused more severe clinical symptoms	(33)
	PRRSV-2: IAF-Klop SS2: P1/7 PRRSV then SS *In vitro*	Cells: MARC-145	- IL-6, CCL5, and TNF-α are synergistically up-regulated in co-infection - An additive effect could be observed for CCL4, CCL14, CCL20, and IL-15	(22)
	PRRSV-2: TJxq1701 SS2: TJ SS then PRRSV *In vivo*	Swine: 3-week-old piglets	- Co-infection caused more severe clinical symptoms and Histopathological damages	(46)
*Actinobacillus pleuropneumoniae*	PRRSV-1: Lelystad APP:1421 PRRSV then APP *In vivo*	Swine: 3-week-old piglets (SPF)	- Co-infection did not cause more severe clinical symptoms - The observation results are different in the acute and subacute phases after co-infection	(86)
	PRRSV-2: IAF-Klop APP: S4074 PRRSV then APP *In vitro*	Cells: MARC-145 Cells: SJPL Cells: PAM	- Bacterial adhesion is not affected by PRRSV - Co-infection produced additive cytotoxic effects - APP showed anti-PRRSV activity	(87)
	PRRSV-2: IAF-Klop APP: MBHPP147 PRRSV then APP *In vitro*	Cells: MARC-145 Cells: SJPL Cells: PAM	- APP anti-PRRSV effect occurs in the early stage of PRRSV infection - Anti-PRRSV may be achieved by affecting PRRSV endocytosis	(88)

### Co-infection of PRRSV and HPS

HPS is a gram-negative bacterium and a common pathogen causing respiratory disease in swine. It can invade, and cause severe systemic disease under suitable conditions, such as fibrinous polyserositis, arthritis, and meningitis (89).

PRRSV infection can predispose swine to secondary infection by destroying PAM cells and inducing inflammation of the nasal mucosa (90). The high detection rate of HPS in PRRSV-infected pig farms implies that PRRSV infection increases the susceptibility for HPS (91). Indeed, studies have demonstrated that PRRSV can increase secondary HPS invasion. However, *in vivo* and *in vitro* studies appear to have produced different results. In 1997, a study of coinfection *in vivo* has reported that there were no more serious clinical symptoms in the co-infection group (84). However, an *in vitro* study showed that a strong pro-inflammatory immune response is triggered by co-infection in PAM cells (85). Besides, HP-PRRSV was found to promote HPS proliferation in blood and tissues in co-infection studies, which could explain the susceptibility of HPS in PRRSV-positive pig populations (92). Further, changes in cytokines have been reported in other studies. It is found that co-infection of PRRSV with HPS can increase the expression of pro-inflammatory cytokines such as TNF-α, IL-1β, and IL-8 in PAM cells. At the protein level, it is also confirmed that co-infection has an additive effect on IL-1β (85). In addition, recent related studies have shown that transfection of HPS RNA enhances HP-PRRSV-mediated inflammatory responses in co-infection (23), which expands on previous studies examining only the effect of PRRSV on HPS. In summary, the co-infection of PRRSV and HPS cannot be explicitly defined as a cooperative promotion, since different results were found in the co-infection group. Moreover, the reasons for these results are closely related to a better immune system *in vivo*. In the current general trend of the epidemic of recombinant PRRSV strains, their high recombination rate and widespread transmission increase the morbidity and mortality of HPS. Therefore, research dedicated to the mechanisms of co-infection between the currently prevalent strains and HPS is more appropriate for the prevention and control of HPS outbreaks.

### Co-infection of PRRSV and SS

SS is a gram-positive bacterium that is believed to be responsible for various clinical disease syndromes in swine (93). SS can be divided into type I and type II. Its symptoms mainly include arthritis, meningitis, pneumonia, septicemia, endocarditis, polyserositis, abortion, and abscess (94). Moreover, SS II can infect humans, causing meningitis, septicemia, and endocarditis (95).

PRRSV and SS coinfection usually causes disease progression, leading to increased morbidity and mortality (87). In the early days, most studies were based on the invasion of individual SS to investigate the pathogenic mechanisms, and the studies on coinfection were scarce. Since 2,000, there has been an increasing number of studies on PRRSV and SS coinfection. Many studies have shown that PRRSV infection can increase susceptibility to SS and cause more severe clinical symptoms (86, 96, 97). One of the studies showed that PRRSV infection suppressed cell immune function of PAM, thereby affecting their ability to clear SS, leading to a wider spread of SS in tissues. Furthermore, it exacerbates the development of related diseases, among which swine inoculated with the HP-PRRSV strain are most severely infected by SS (86). Another study reported the expression levels of inflammatory factors in bone marrow dendritic cells (BMDCs) infected by the two pathogens. The results showed that secondary infection of SS after PRRSV infection resulted in an additive effect of CCL4, CCL14, CCL20, and IL-15, with significant synergistic upregulation of IL-6, CCL5, and TNF-α (87). There are many studies with similar results, suggesting that secondary infection with SS enhances the inflammatory response mediated by PRRSV infection (97, 98). Furthermore, recent studies have reported that early infection with SS type II increased the virulence of the HP-PRRSV MLV-like strain (TJxq1701), causing an excessive inflammatory response and tissue damage, resulting in higher morbidity and mortality in piglets (88). It is suggested that recombination between PRRSV modified live vaccine strains and other PRRSV strains leads to the emergence of PRRSV MLV-like strains and secondary infection with SS causes more severe clinical signs. Also, there appears to be a cooperative promotion between PRRSV and SS. This provides a reference for the prevention and control of PRRSV as well as coinfection studies.

### Co-infection of PRRSV and APP

APP is a gram-negative bacterium which can cause highly contagious respiratory disease (99). APP can be broadly classified into two types, namely, biotype 1 and biotype 2. According to the polysaccharide antigen on its surface, biotype 1 has 12 serotypes, and biotype 2 has 6 serotypes (100). Swine of all ages can be infected with APP; however, the clinical symptoms are different according to their APP strains and infection cycle (101).

As early as 1997, an *in vivo* study was carried out on the coinfection of PRRSV and APP. It was found that the experimental results were completely inconsistent between the acute and subacute stages after co-infection. It was observed that secondary infection with APP in the acute phase could lead to more severe disease, but it did not have any effect during the subacute phase (33). This is consistent with results from another *in vitro* study, in which pre-infection with PRRSV did not affect the adhesion capacity of APP (22). In addition, other studies have found that pre-infection of *in vitro* cell lines with APP, completely blocked PRRSV infection. Even the supernatant of APP cultures was sufficient to significantly block PRRSV infection, presumably in association with heat-resistant APP metabolites (22). Further, its anti-PRRSV mechanism has been investigated in recent studies. It is found that this blocking effect may be achieved by activating cofilin and causing actin depolymerization, which in turn affects PRRSV endocytosis (46). In summary, PRRSV had little effect on APP while APP possessed anti-PRRSV infection activity, suggesting an inhibitory effect between them. However, studies on the underlying molecular mechanisms do not seem to have explored in depth the molecular associations between the two pathogens, such as how APP metabolites are associated with cofilin in PRRSV. Such a problem seems to be a limitation in every coinfection study. Thus, whether future studies can find prevention and control strategies based on the molecular link between the two pathogens remains to be further investigated.

## Coinfection With Other Pathogens

In addition to coinfection with the above-mentioned viruses and bacteria, PRRSV can co-infect with other pathogens, such as *T. gondii*, Mhp. *T. gondii* is a specific intracellular protozoan parasite that can invade any nucleated host cell (102). Among these, the pig is one of the intermediate hosts. *T. gondii* can also cause opportunistic pathogen infection (103). PRRSV is a common pathogen that causes coinfection with *T. gondii*. Through the evaluation of 372 samples from 9 provinces in China, it was found that the coinfection rate was 1.61% (26). This is the first time that coinfection of PRRSV and *T. gondii* has been reported in China, but there is no further explanation of the pathological manifestations after co-infection. A case of co-infection was also found in South Korea. The pathological manifestations in the lungs were alveolar macrophage necrosis, hemorrhage, multiple necrosis, and diffuse interstitial pneumonia. It was found that *T. gondii* infection can lead to more serious pathological changes, and it is speculated that *T. gondii* promotes PRRSV infection. However, there are few studies on parasites and PRRSV, especially on the related mechanisms.

Mhp infection can destroy the ciliated epithelium of the respiratory tract, thereby adversely affecting the mucosal system. The main clinical symptoms are chronic cough, and coinfection is often accompanied by other clinical symptoms such as fever and growth impairment. Mhp is also one of the main causes of PRDC, which is often isolated with PRRSV. It is suggested that Mhp plays an important role in PRRSV infections. Studies on related coinfection have been conducted for a long time. Some studies have pointed out that Mhp infection can increase the production of pro-inflammatory factors, which in turn leads to an increase in the incidence of PRRSV (25). Through bioinformatics analysis of differential genes in PAM cells after coinfection, some researchers found that inflammatory factors such as CCL-4, IL-1β, IL-1α, and CCL-2 were significantly up-regulated after coinfection (104). It is suggested that excessive inflammatory reaction is one of the causes of severe lung lesions after coinfection, which confirms the results of previous studies (105). This provides a reference for the study of the mechanism of coinfection and acts as a guide for the prevention and control of PRDC.

## Perspectives

As shown in this review, the emergence of new strains such as NADC30-like, represents a high mutation stage of the PRRSV epidemic in China, and PRRSV-2 appears to become more common (106, 107). Not only the NADC30-like strain itself is highly recombinant, but its coinfection with other pathogens increased the mutation rate of PRRSV. This affects the molecular outcome of the virus itself and the host, such as the evolution of the virus, clinical symptoms, susceptibility to antiviral therapy, and lethality of the virus infecting the host. As one of the main strains in the current epidemic, HP-PRRSV is also susceptible to coinfection and recombination with other strains (10). The continuous evolution of PRRSV has made the study of pathogenic mechanisms strenuous as well as the clinical signs of PRRS and co-infection with other porcine pathogens difficult to diagnose. Therefore, in addition to the study of the pathogenic mechanism, it is necessary to control the virus mutation caused by co-infection.

**Figure 1:**
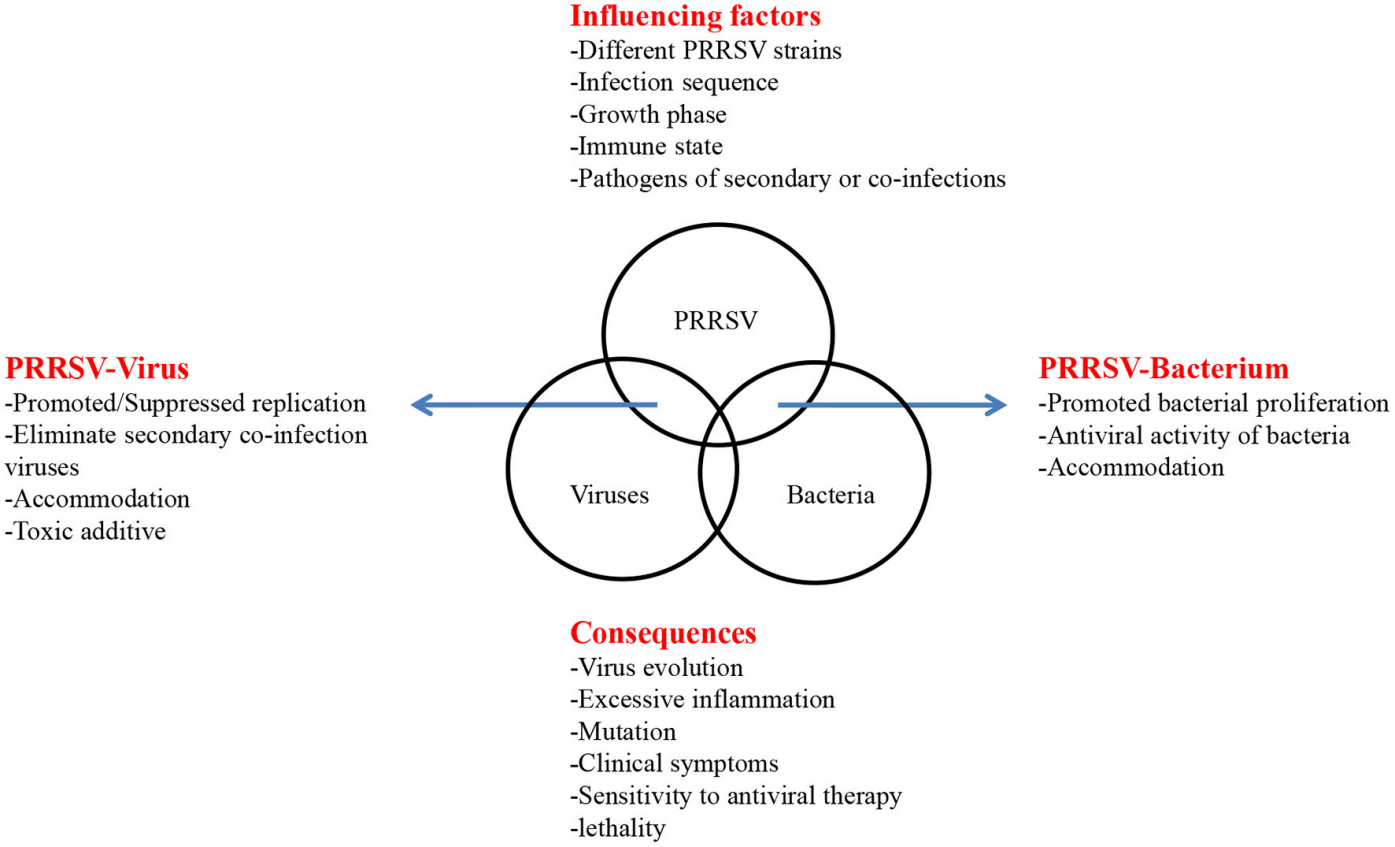
Influencing factors and consequences of PRRSV co-infection. The interaction of coinfection is listed by arrows on both sides. In the top box, factors that may affect coinfections are listed. In the below box, some consequences of coinfections are listed.

However, the high mutation rate of PRRSV, the variable epidemiological trends, the complexity of the interactions among different PRRSV strains and other porcine pathogens, the different molecular consequences of co-infection, and numerous abiotic factors limit the study of co-infection. Moreover, after reviewing the above *in vivo* and *in vitro* experiments and field co-infection rate, we briefly summarized the interactions, influencing factors and molecular consequences between PRRSV and other porcine pathogens (Figure 1). Different strains, infection sequence, cell selection, animal selection, immune status and individual differences, and even pig sex and different ages may cause the different co-infection results. Further, the unclear pathogenic mechanism of single virus also greatly limits the relevant co-infection research. Therefore, in addition to studying the interactions between PRRSV and other porcine pathogens during co-infection, more research is needed on how these alter the host immune response and influence the effectiveness of vaccination. Hence, further research on the recombination of PRRSV is warranted. These studies may provide a new strategy for the prevention and control of PRRSV co-infection associated diseases.

## Author Contributions

DZhao, BY, and KZ conceived and designed the study. DZhao and XL wrote the original draft of the manuscript. KZ, HZ, XY, CS, DZhan, XS, TZ, HC, XC, and YH wrote sections of the manuscript. All authors contributed to the article and approved the submitted version.

## Conflict of Interest

The authors declare that the research was conducted in the absence of any commercial or financial relationships that could be construed as a potential conflict of interest.

## Publisher's Note

All claims expressed in this article are solely those of the authors and do not necessarily represent those of their affiliated organizations, or those of the publisher, the editors and the reviewers. Any product that may be evaluated in this article, or claim that may be made by its manufacturer, is not guaranteed or endorsed by the publisher.
